# Comparison of Ondansetron and Cyclizine for the Management of Postoperative Nausea and Vomiting: A Systematic Review and Meta-Analysis

**DOI:** 10.7759/cureus.101982

**Published:** 2026-01-21

**Authors:** Shahmeer Hamid, Badr Bahaj, Zain U Sajhad

**Affiliations:** 1 Medicine, Bedfordshire Hospitals NHS Foundation Trust, Luton, GBR; 2 Emergency Medicine, Queen Alexandra Hospital, Portsmouth, GBR; 3 School of Medicine, University of Birmingham, Birmingham, GBR

**Keywords:** antiemetic, cyclizine, day care surgery, nausea and vomiting, ramosetron and ondansetron

## Abstract

Postoperative nausea and vomiting (PONV) are frequent complications following general anaesthesia, demanding effective and cost-efficient prophylactic strategies. Therefore, this study aimed to assess the comparative efficacy and safety of ondansetron versus cyclizine for PONV prophylaxis in adult surgical patients by adopting a meta-analysis research approach. The Preferred Reporting Items for Systematic Review and Meta-Analysis (PRISMA) guidelines were adopted in selecting and screening the studies. A computer-based search of the EMBASE, MEDLINE, CENTRAL, and CINAHL databases was carried out using the last search up to November 2025. The risk of bias of the included randomised controlled trials (RCTs) was assessed using the Cochrane Risk of Bias 2 (RoB 2) tool. A sensitivity analysis was performed to assess robustness, and the certainty of evidence was evaluated using Grading of Recommendations Assessment, Development and Evaluation (GRADE). All statistical analyses were performed using MetaAnalysisOnline software. Four RCTs involving 433 adult surgical patients were included. Pooled analysis showed that ondansetron did not significantly reduce the incidence of any PONV compared to cyclizine (odds ratio: 0.74, 95% CI: 0.34 to 1.61, p=0.45). Sensitivity analysis confirmed this finding across surgical subtypes. Additionally, there were no significant differences in the incidence of postoperative vomiting (odds ratio: 1.11, 95% CI: 0.61 to 2.02, p=0.74) or the requirement for rescue antiemetics (odds ratio: 1.83, 95% CI: 0.95 to 3.52, p=0.07). However, moderate heterogeneity was observed (I^2^=68%) for the primary outcome, and cyclizine was associated with a statistically significant, though clinically small, delay in time to eye opening (mean difference: 2.00 min, p<0.001). The overall certainty of evidence was graded as low to very low. Low-certainty evidence indicates that cyclizine is a reasonable approach to preventing PONV, offering comparable efficacy to ondansetron without prolonging hospital discharge times. Due to its cost-effectiveness and potential specific benefit in diagnostic laparoscopy, it might be useful for ambulatory surgical centres aiming to optimize value-based care. However, the efficacy of cyclizine within modern multimodal protocols has not been consistently determined by these monotherapy trials, and long-term assessments of combined antiemetic strategies need to be evaluated.

## Introduction and background

Postoperative nausea and vomiting (PONV) are common complications following surgery under general anaesthesia, affecting approximately 30% of all surgical patients and up to 80% of high-risk individuals [1]. Beyond the immediate degradation of patient comfort, PONV is associated with tangible clinical sequelae, including aspiration pneumonitis, surgical wound dehiscence, and electrolyte imbalances [2]. Furthermore, it represents a significant economic burden, often leading to prolonged recovery room stays and unanticipated hospital admissions following ambulatory surgery [1,3]. To mitigate these risks, current consensus guidelines advocate for multimodal prophylaxis targeting different receptor pathways within the chemoreceptor trigger zone (the area of the medulla that receives emetic stimuli) and the vomiting centre [1].

Multiple neurotransmitter pathways are implicated in the pathophysiology of PONV, including serotonergic, dopaminergic, histaminergic, and muscarinic systems [4]. Ondansetron, a selective 5-hydroxytryptamine-3 (5-HT3) receptor antagonist, has become one of the most widely used antiemetics due to its proven efficacy and favourable side-effect profile [5,6]. However, its use has historically been constrained by higher costs and specific side effects such as headache and QTc prolongation [1,7]. Conversely, cyclizine, a histamine (H1) receptor antagonist with anticholinergic properties, offers a significantly lower-cost alternative that has been a staple of anaesthetic practice for decades, particularly in the United Kingdom [8,9]. While effective, cyclizine is often associated with sedation, tachycardia, and pain on injection, leading to debate regarding its suitability for ambulatory surgery [9-11].

Despite the routine use of both agents, clinical decision-making is often driven by institutional habit or cost considerations rather than definitive comparative evidence. While recent large-scale network meta-analyses (which compare multiple treatments simultaneously using both direct and indirect evidence) have established that both drugs are superior to placebo [12], direct head-to-head comparisons have yielded conflicting results regarding their relative efficacy. For instance, early randomised controlled trials (RCTs) by Watts observed a distinct superiority of ondansetron over cyclizine, reporting significantly lower nausea scores and higher efficacy in the ondansetron group [10]. In contrast, subsequent studies by Grimsehl et al. and Cholwill et al. found the two agents to be clinically equivalent, arguing that the significantly lower cost of cyclizine made it the preferable first-line agent [8,11].

To date, no dedicated systematic review and meta-analysis has exclusively synthesised the direct randomised evidence comparing ondansetron and cyclizine. Existing reviews often rely on indirect comparisons derived from network meta-analyses or group cyclizine with other antihistamines, potentially obscuring distinct differences in side-effect profiles and recovery metrics [12]. Given the ongoing need for cost-effective, evidence-based anaesthetic practice, a quantitative synthesis of direct comparative trials is warranted to resolve these discrepancies.

The primary objective of this systematic review and meta-analysis is to evaluate the comparative efficacy of ondansetron versus cyclizine for PONV prophylaxis in adult surgical patients. Specifically, this review aims to quantify the difference in the incidence of any PONV, vomiting, and the requirement for rescue antiemetics between the two groups, analyse the safety profile of each agent, and assess secondary recovery outcomes, including time to eye opening and patient satisfaction, to determine if the use of cyclizine results in delayed recovery compared to ondansetron.

## Review

Methods

Search Strategy

The Preferred Reporting Items for Systematic Reviews and Meta-Analyses (PRISMA) framework was utilised for this systematic review and meta-analysis [13]. A review protocol was not prospectively registered for this study due to the rapid timeline of the review process.

Embase, MEDLINE, CENTRAL, and CINAHL databases were systematically searched in November 2025 to comprehensively identify English-language, peer-reviewed literature, with no limits on publication date or country of publication to ensure maximal study inclusion. The search included free-text keywords and controlled vocabulary (e.g., MeSH and Emtree terms) tailored to each database’s specific syntax. The following search terms were included: "ondansetron", "cyclizine", "postoperative nausea and vomiting", "postoperative vomiting", "PONV", "randomized controlled trial", "randomized", and "trial". The full search strategies for all databases are detailed in Appendix Table 4 of this review. Furthermore, any additional studies eligible for review were captured by screening the references of previously identified studies [14].

Inclusion and Exclusion Criteria

The selection and screening of research articles were guided by pre-defined eligibility criteria. Studies that satisfied the following inclusion criteria were included: (i) adult patients (aged ≥18 years) undergoing any type of surgical procedure under general or regional anaesthesia; (ii) prophylactic administration of ondansetron at the time of anaesthetic induction via any route (IV, intramuscular (IM), oral, sublingual); (iii) prophylactic administration of cyclizine at the time of anaesthetic induction via any route; (iv) studies reporting at least one primary or secondary outcome of interest, including the incidence or severity of nausea and vomiting postoperatively, rescue antiemetic requirements, adverse events, or postoperative recovery metrics (e.g., time to eye opening, time to discharge); (v) RCTs, controlled clinical trials, or quasi-randomised studies; and (vi) articles published in English.

The following exclusion criteria were applied: (i) procedures performed under sedation without general or regional anaesthesia; (ii) protocols involving the concurrent use of other prophylactic antiemetics (multimodal antiemesis) that would confound the direct comparison; (iii) studies failing to report specific PONV event rates or relying solely on surrogate endpoints (e.g., nausea scores without incidence data); (iv) non-comparative study designs, including case reports, case series, reviews, editorials, animal studies, and in vitro research; and (v) non-English language articles where a translation was unavailable.

Study Selection Process

Independent screening of titles and abstracts was conducted by two reviewers (SH and BB) using the software Rayyan (Rayyan Systems, Inc., Cambridge, MA). Full-text eligibility was assessed independently in accordance with the inclusion and exclusion criteria, with disagreements resolved via adjudication by a third reviewer (ZUS).

Data Extraction 

A data extraction spreadsheet was developed using Google Sheets and included the following information: author, publication year, study design, number of patients randomised to each treatment group, sex distribution, mean age, mean body mass index (BMI), surgical procedure, intervention, anaesthetic type, mean anaesthesia duration, mean surgery duration, last follow-up, primary outcome(s), and secondary outcomes. Data extraction was performed collaboratively, with disagreements resolved through discussion among the authors. 

The certainty of the evidence was assessed using the Grading of Recommendations Assessment, Development and Evaluation (GRADE) approach, categorising evidence as high, moderate, low, or very low quality based on risk of bias, inconsistency, indirectness, imprecision, and publication bias [15].

Risk-of-Bias Assessment

The risk of bias in the included RCTs was evaluated using the Cochrane Risk of Bias 2 (RoB 2) tool [16]. In accordance with Cochrane guidance, study quality was assessed across five domains: the randomisation process, deviations from intended interventions, missing outcome data, measurement of the outcome, and selection of the reported result. Each study was independently appraised by two reviewers (SH and BB), categorising the risk of bias within each domain, and as an overall judgement, as 'low risk', 'some concerns', or 'high risk'.

Statistical Analysis

MetaAnalysisOnline software (https://metaanalysisonline.com) was used to conduct all statistical analyses for the study [17]. Outcomes that were reported in fewer than three studies were analysed qualitatively as secondary outcomes. Continuous variables were assessed by the mean difference, whilst dichotomous variables were assessed by the odds ratio (OR) where applicable. MetaAnalysisOnline was used to produce forest plots with 95% confidence intervals (CI) and determine heterogeneity. Pooled analyses were conducted using random-effects models to account for anticipated between-study heterogeneity; a p-value of <0.05 was classified as statistically significant. Assessment of publication bias using funnel plots was not performed due to the limited number of included studies (n<10), in accordance with the standard recommendations for meta-analyses. A sensitivity analysis was conducted by excluding the study involving laparoscopic cholecystectomy (Malak et al. [9]) to assess the consistency of results across surgical subtypes, specifically gynaecological laparoscopy.

The heterogeneity for studies included in the meta-analysis was calculated using the I^2^ statistic [18]. This was interpreted according to the following scale: 0-24% represented low heterogeneity, 25-74% represented moderate heterogeneity, and values ranging from 75-100% were deemed as having high heterogeneity.

Results

Literature Search Results

**Figure 1:**
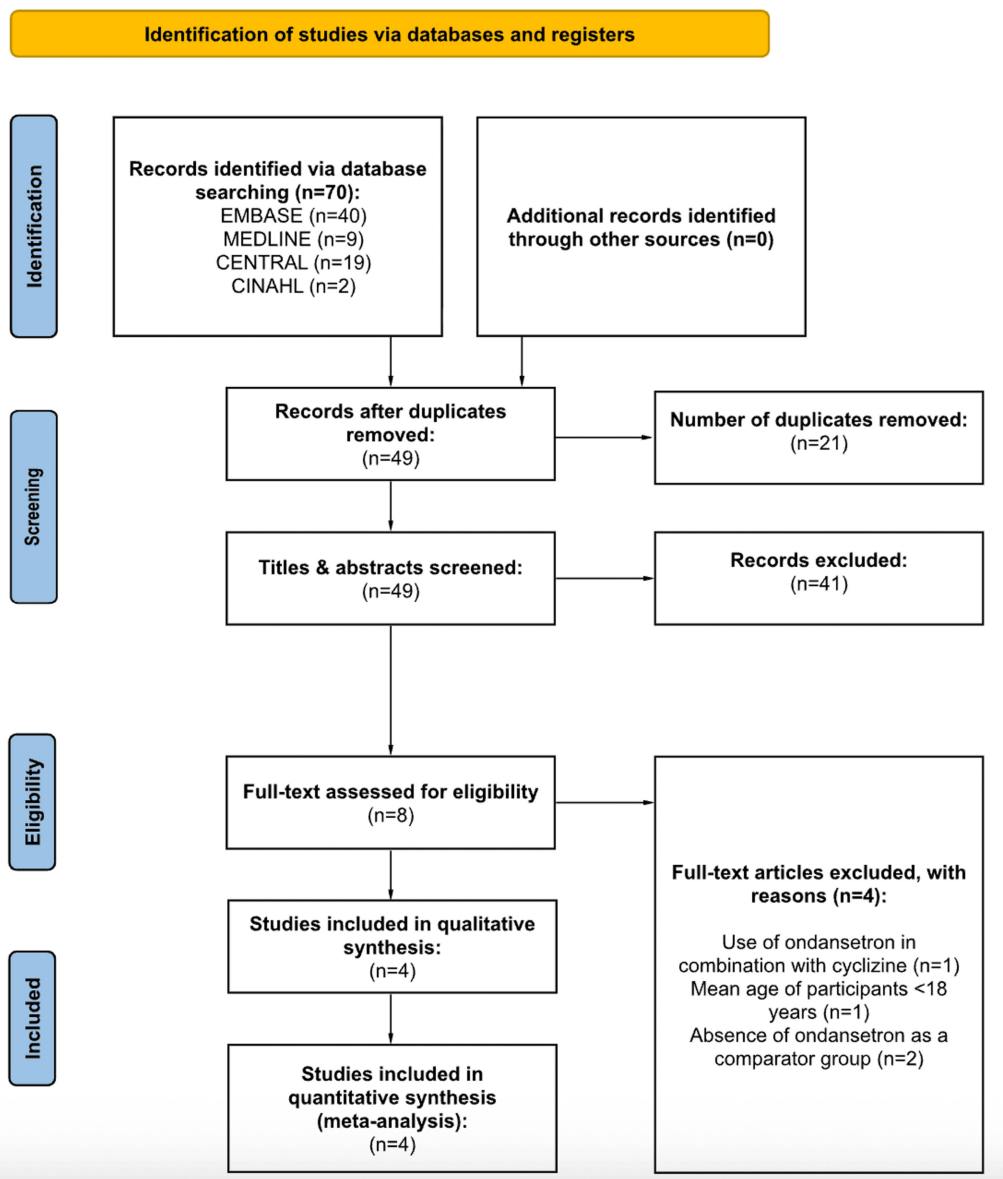
PRISMA flow diagram for the screening and selection of studies. PRISMA: Preferred Reporting Items for Systematic Reviews and Meta-Analyses Ref. [13].

A total of 70 articles were reviewed by two independent authors through the online search of Embase, MEDLINE, CENTRAL, and CINAHL. Following the removal of duplicates and initial screening, studies were excluded for the following reasons: use of ondansetron in combination with cyclizine, mean age of participants being under 18 years, and absence of ondansetron as a comparator group. Four RCTs met the eligibility criteria upon full-text review and were included in the quantitative synthesis (Figure 1).

Study Characteristics

This systematic review and meta-analysis included four RCTs comparing ondansetron and cyclizine for the prevention of PONV [8-11]. The principal characteristics of the included studies are summarised in Table 1. All trials were prospective, double- or single-blinded randomised studies published between 1996 and 2017. Across the four studies, a total of 433 participants undergoing elective surgical procedures under general anaesthesia were analysed, predominantly involving women undergoing day-case or short-stay laparoscopic surgery. Sample sizes of individual trials ranged from 74 to 130 participants. The mean age of participants and BMI ranged from 30.4 to 47.5 years and 23 to 26.3, respectively. Follow-up durations were short and clinically relevant, extending from the immediate postoperative period through to 24 hours after surgery.

**Table 1:** Characteristics of included studies. RCT: randomised controlled trial; SD: standard deviation; IV: intravenous; GA: general anaesthesia; NR: not recorded; PONV: postoperative nausea and vomiting.

Author (Year)	Study Design	N (Ondansetron)	N (Cyclizine)	Female Sex, n (%)	Mean Age (SD)	Mean BMI (SD)	Surgical Procedure	Intervention	Anaesthetic Type	Mean Anaesthesia Duration (SD)	Mean Surgery Duration	Last Follow-up	Primary Outcome(s)	Secondary Outcome(s)
Watts (1996) [10]	Double-blind RCT	59	53	112 (100%)	30.4 (6.3)	NR	Gynaecological Laparoscopy (Diagnostic & Sterilisation)	Ondansetron 4 mg IV; Cyclizine 50 mg IV	GA	NR	NR	24 hours postoperatively	Clinically significant PONV (nausea score ≥2).	Nausea severity scores, sedation scores, morphine requirement, admission, adverse effects (injection pain, rash)
Cholwill et al. (1999) [11]	Double-blind RCT	60	57	117 (100%)	31.5 (5.5)	23.0 (2.5)	Gynaecological Laparoscopy (Diagnostic & Sterilisation)	Ondansetron 4 mg IV; Cyclizine 50 mg IV	GA	NR	NR	24 hours post-discharge	Incidence of moderate-to-severe PONV.	Rescue antiemetic requirement, sedation scores, postoperative pain scores, postoperative analgesia use.
Grimsehl et al. (2002) [8]	Double-blind RCT	37	37	74 (100%)	32 (5.6)	24.8 (4.6)	Gynaecological Laparoscopy (Diagnostic & Sterilisation)	Ondansetron 4 mg IV; Cyclizine 50 mg IV	GA	Ondansetron: 25 min (9); Cyclizine: 26 min (11)	NR	24 hours post-discharge	Incidence and severity of PONV.	Time to eye opening, time to discharge, rescue antiemetic requirement, incidence of postoperative pain, sedation requirement, analgesia requirement.
Malak et al. (2017) [9]	Single-blind RCT	65	65	97 (74.6%)	47.5 (8.3)	26.3 (4.0)	Laparoscopic cholecystectomy	Ondansetron 4 mg IV; Cyclizine 50 mg IV	GA	Ondansetron: 104.6 min (6.6); Cyclizine: 106.8 min (7.5)	Ondansetron: 84.6 min (6.6); Cyclizine: 83.2 min (6.5)	24 hours postoperatively	Incidence of PONV (within 24 h).	Rescue antiemetic requirement, adverse effects (headache, dizziness, sedation).

Table 2 provides a summary of the key results from the four RCTs included in this study [8-11].

**Table 2:** Summary of results from the included studies. PONV: postoperative nausea and vomiting; NS: not significant.

Author (Year)	Summary of Key Results
Watts (1996) [10]	Primary: Ondansetron was superior, with significantly lower rates of clinical PONV (score ≥2) compared to cyclizine (20% vs 51%, p=0.03); Secondary: Nausea scores were significantly lower for ondansetron at 2 h (p=0.008) and discharge (p=0.002). Sedation scores, admission rates, and morphine requirements were comparable (p=NS).
Cholwill et al. (1999) [11]	Primary: No overall difference in pre-discharge vomiting (30% vs 23%) or rescue antiemetic use (28% vs 16%). Post-discharge vomiting rates were also similar (28% vs 21%); subgroup analysis: In diagnostic laparoscopy specifically, cyclizine was superior, with significantly lower rescue antiemetic requirements (37% vs 4%, p<0.01) and reduced vomiting (37% vs 13%, p=0.05).
Grimsehl et al. (2002) [8]	Primary: No difference in total PONV at 24 h (54% vs 56%), pre-discharge nausea VAS scores showed no significant difference (39±26 vs 49±24). Secondary: Patients in the cyclizine group took significantly longer to open eyes (10±4 min vs 8±2 min, p<0.001), but time to discharge was unaffected (309 min vs 324 min, p=NS), rescue antiemetic requirement was similar (14% vs 11%).
Malak et al. (2017) [9]	Primary: No significant difference in PONV incidence (nausea: 7.7% vs 4.6%, p=0.67; vomiting: 9.2% vs 10.8%, p=0.67). Secondary: Rescue antiemetic requirement was similar (10.8% vs 6.2%, p=0.72). No significant differences in headache (p=0.79), dizziness (p=0.63), or sedation (p=0.91).

Risk-of-Bias Assessment

**Figure 2:**
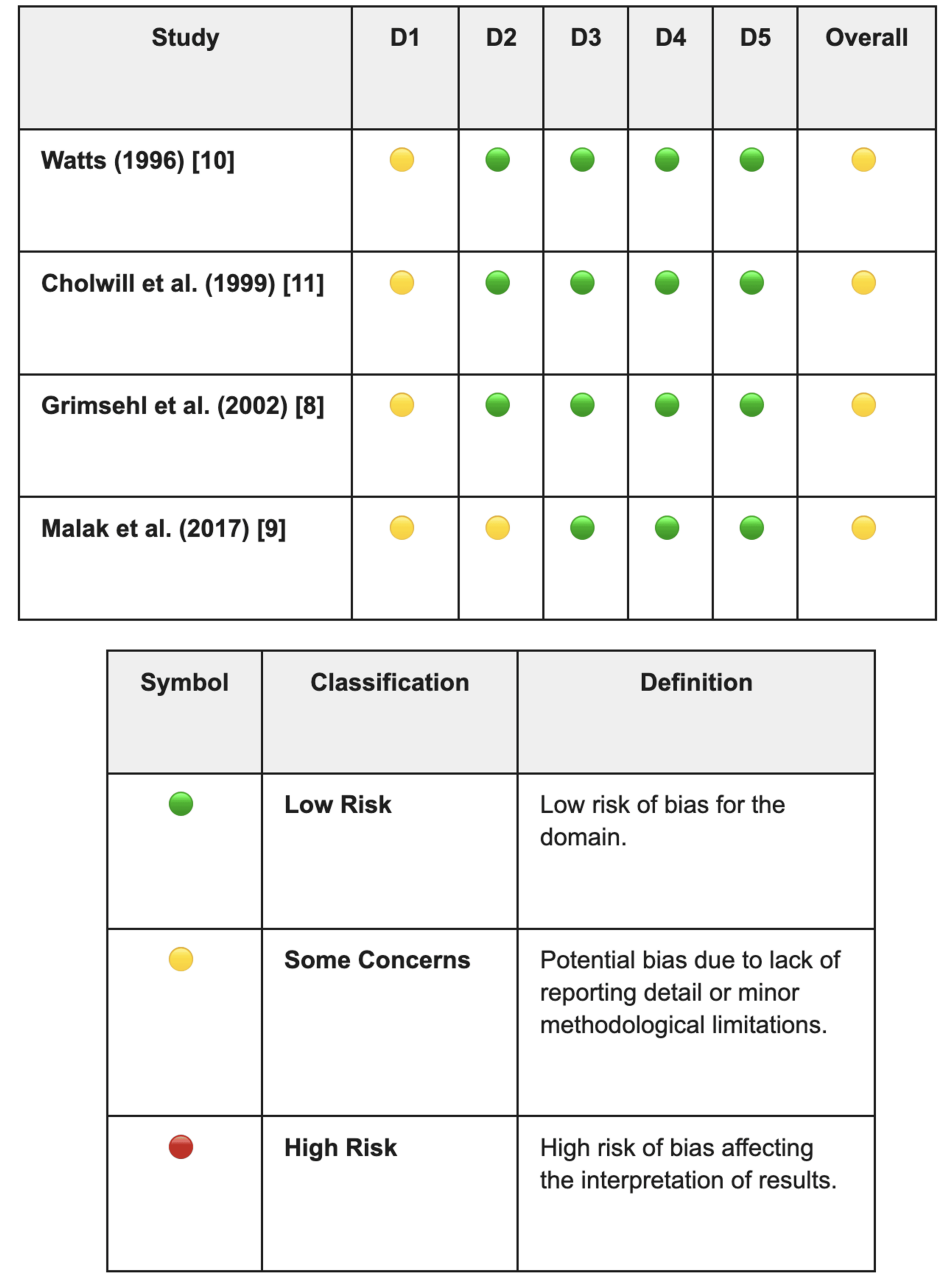
Cochrane Risk of Bias 2 (RoB 2) assessment of included randomised controlled trials. D1: bias arising from the randomisation process; D2: bias due to deviation from the intended interventions; D3: bias due to missing outcome data; D4: bias in measurement of the outcome; D5: bias in selection of the reported result [16]. Refs. [8-11].

Applying the Cochrane RoB 2 tool, as demonstrated in Figure 2, to the four included trials resulted in a uniform classification of 'some concerns' [8-11]. This rating largely reflects gaps in documentation rather than fundamental methodological flaws in study design. Although all studies implemented randomisation, they often failed to detail the specific methods used for sequence generation or allocation concealment, which necessitated a cautious rating in the randomisation domain. In terms of protocol adherence, the risk of deviation was generally minimised through double-blind designs. The notable exception was the study by Malak et al. [9], which was flagged with 'some concerns' due to its single-blind methodology; because the administering anaesthetist was aware of the treatment allocation, a theoretical risk of performance bias exists. Despite these issues, the reliability of the data remained high. Attrition was negligible, effectively neutralising the risk of missing data, and the objectivity of outcome measurement was preserved by using blinded assessors, such as recovery ward nurses. Furthermore, there was no evidence of selective reporting, as all anticipated outcomes were fully disclosed.

Primary outcomes

*Incidence of Any PONV Within 24 h or Study Period*

**Figure 3:**
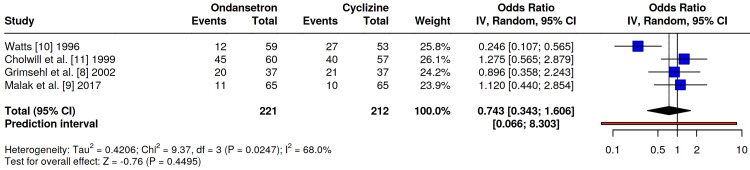
Odds ratio comparing the incidence of any PONV within 24 h or study period between ondansetron and cyclizine groups. No significant difference was seen: OR 0.74, 95% CI 0.34-1.61, p=0.45. PONV: postoperative nausea and vomiting. Refs. [8-11].

All four included studies, Grimsehl et al. [8], Malak et al. [9], Watts [10], and Cholwill et al. [11], evaluated the overall incidence of PONV between ondansetron and cyclizine. Three investigations demonstrated comparable efficacy between the two antiemetics; however, one study [10] observed a significantly lower incidence of PONV in the ondansetron group. A pooled analysis revealed no statistically significant difference in overall PONV rates between the two groups (OR 0.74, 95% CI 0.34-1.61, p=0.45). Notably, moderate statistical heterogeneity was observed (I²=68%) (Figure 3), likely reflecting the outlier results reported in the Watts study [10] compared to the more homogeneous findings of the other three trials.

**Figure 4:**
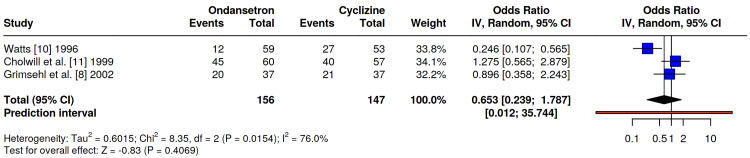
Forest plot of the sensitivity analysis comparing the incidence of PONV (within 24 h or study period) between ondansetron and cyclizine in patients undergoing laparoscopic gynaecological surgery. No significant difference was observed: OR 0.65, 95% CI 0.24-1.79, p = 0.41. The analysis includes only studies involving gynaecological laparoscopy. No significant difference was observed between ondansetron and cyclizine. Malak et al. [9] was excluded from this sensitivity analysis as this involved patients undergoing laparoscopic cholecystectomy. Refs. [8,10,11].

A sensitivity analysis excluding the study by Malak et al. [9] (laparoscopic cholecystectomy) was performed to isolate the effects in gynaecological laparoscopy. The exclusion of this study did not alter the overall findings, as the remaining gynaecological studies maintained the previously observed heterogeneity, driven primarily by Watts [10]. A pooled analysis (Figure 4) showed no statistically significant difference in overall PONV rates between the two groups (OR 0.65, 95% CI 0.24-1.79, p=0.41). Notably, a high statistical heterogeneity was observed (I²=76%). 

Incidence of Postoperative Vomiting

**Figure 5:**
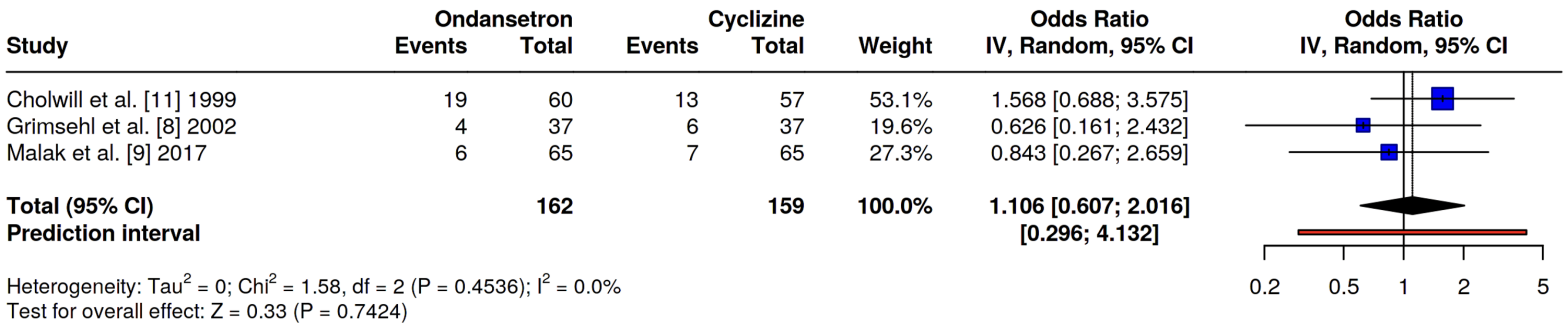
Odds ratio comparing the incidence of postoperative vomiting between ondansetron and cyclizine groups. No significant difference was seen: OR 1.11, 95% CI 0.61-2.02, p=0.74. Refs. [8,9,11].

Three studies, Grimsehl et al. [8], Malak et al. [9], and Cholwill et al. [11], compared the specific incidence of postoperative vomiting episodes between the ondansetron and cyclizine cohorts. Across these investigations, the frequency of vomiting remained comparable between the groups; however, one study [11] identified a non-significant trend toward fewer emetic episodes in the cyclizine group. A pooled analysis confirmed no statistically significant difference in overall postoperative vomiting rates between the two agents (OR 1.11, 95% CI 0.61-2.02, p=0.74), with no evidence of statistical heterogeneity (I²=0%) (Figure 5).

*Requirement for Rescue Antiemetics*

**Figure 6:**
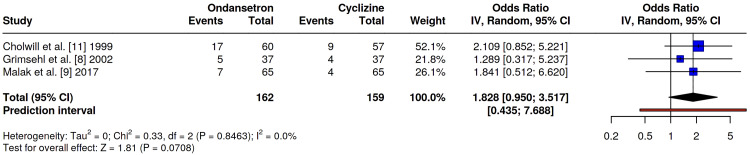
Odds ratio comparing the requirement for rescue antiemetics between ondansetron and cyclizine groups. No significant difference was seen: OR 1.83, 95% CI 0.95-3.52, p=0.07. Refs. [8,9,11].

The requirement for rescue antiemetic therapy was compared across three studies: Grimsehl et al. [8], Malak et al. [9], and Cholwill et al. [11]. Across these studies, patients receiving cyclizine required similar or fewer rescue interventions compared to those in the ondansetron cohorts. Notably, Cholwill et al. [11] reported a lower absolute requirement for rescue medication in the cyclizine group following diagnostic laparoscopy. However, pooled analysis demonstrated no statistically significant difference in rescue antiemetic requirements between the two groups (OR 1.83, 95% CI 0.95-3.52, p=0.07), with low heterogeneity (I²=0%) (Figure 6).

Secondary outcomes

Nausea Severity

Three studies, Cholwill et al. [11], Grimsehl et al. [8], and Watts [10], assessed the severity of nausea using varying scoring systems (Linear Analogue Scales or categorical severity grades). Cholwill et al. [11] found that while both drugs significantly reduced moderate-to-severe nausea compared to placebo (ondansetron 30% and cyclizine 23% vs. placebo 52%, p=0.02 and p=0.001, respectively), there was no significant difference between ondansetron and cyclizine themselves in preventing these symptoms (p=0.1 overall). Grimsehl et al. [8] corroborated this, reporting no significant difference in mean nausea scores between the two groups (ondansetron: 39 mm, cyclizine: 49 mm, p=not significant) or in the incidence of severe nausea (ondansetron 16% vs. cyclizine 16%, p=not significant). In contrast, Watts [10] observed a distinct difference; because cyclizine failed to demonstrate efficacy in that specific study (with PONV rates similar to placebo), the mean nausea scores at 2 h and discharge were significantly lower in the ondansetron group compared to the cyclizine group (mean rank: ondansetron 74.9 vs. cyclizine 96.0, p=0.008), reflecting the study's overall finding of ondansetron superiority.

Reported Adverse Effects

Three studies, Malak et al. [9], Watts [10], and Cholwill et al. [11], assessed the incidence of adverse effects, specifically sedation, headache, and dizziness, between ondansetron and cyclizine. Across these studies, the safety profiles were comparable, with no statistically significant differences found in the occurrence of side effects. For sedation, Malak et al. reported a negligible difference (OR 1.52, 95% CI 0.25-9.37, p=1.00) [9], while Cholwill et al. similarly found no significant difference in moderate or severe sedation rates (OR 0.93, 95% CI 0.43-2.03, p=0.86) [11]. Regarding specific neurological symptoms, Malak et al. found no significant difference in the incidence of headache (OR 1.00, 95% CI 0.19-5.15, p=1.00) or dizziness (OR 0.49, 95% CI 0.09-2.76, p=0.68) between the groups [9]. These findings are supported by Watts, who qualitatively reported that the incidence of side effects was low and unremarkable across all treatment arms, with no clinically relevant differences observed between the ondansetron and cyclizine cohorts [10].

Recovery Outcomes

One study, Grimsehl et al. [8], examined recovery times, focusing on how long it took for patients to open their eyes and when they were ready for hospital discharge. They found that patients given cyclizine took slightly longer to open their eyes (10±4 min) compared to those given ondansetron (8±2 min), a difference that was statistically significant (mean difference +2.00 min, 95% CI 0.60-3.40, p<0.001). However, this brief delay did not keep patients in the hospital any longer; there was no significant difference in the total time to discharge between the cyclizine (309±49 min) and ondansetron (324±68 min) groups (mean difference -15.00 min, 95% CI -42.40 to 12.40, p=0.28). This suggests that while cyclizine might cause some initial drowsiness, it does not prolong the overall hospital stay.

Pain on Injection

Watts [10] evaluated the frequency of injection-site discomfort and found it to be a rare occurrence for both treatment arms. Specific reports of pain were minimal, affecting only two patients receiving ondansetron and a single patient receiving cyclizine. Consequently, statistical analysis showed no significant disparity in risk between the two agents (OR 1.81, 95% CI 0.16-20.46, p=1.00). The authors further contextualised this by noting that significant injection pain was primarily observed with metoclopramide, rather than the drugs being compared in this specific analysis.

Cost-Effectiveness

Two studies [8,10] provided a qualitative comparison of the cost-effectiveness of ondansetron versus cyclizine. Both studies consistently identified cyclizine as the more cost-effective option, highlighting a substantial price differential at the time of publication. Grimsehl et al. noted a "10-fold increase in cost" for ondansetron compared to cyclizine, concluding that cyclizine should be the first-choice agent given the comparable efficacy [8]. Similarly, Watts described ondansetron as "considerably more expensive" than the alternatives available, supporting the use of less-expensive agents as first-line prophylaxis [10].

Overall Patient Satisfaction

Two studies, Grimsehl et al. [8] and Cholwill et al. [11], explicitly evaluated patient satisfaction with their postoperative antiemetic control. Both studies found high levels of satisfaction with no statistically significant difference between the ondansetron and cyclizine groups. Cholwill et al. reported that the proportion of patients rating their emetic control as "excellent" or "good" was comparable between ondansetron (82%) and cyclizine (79%) (OR 1.19, 95% CI 0.47-3.02, p=0.71) [11]. Similarly, Grimsehl et al. found that despite the difference in eye-opening times, overall patient satisfaction scores were nearly identical, with 89% of the ondansetron group and 86% of the cyclizine group rating their satisfaction as "good" (OR 1.29, 95% CI 0.32-5.23, p=0.71) [8].

Certainty of evidence (GRADE)

The certainty of evidence was systematically evaluated for all primary and secondary outcomes, resulting in an overall classification of low to very low quality. This grading was primarily influenced by three key factors: the assignment of a "some concerns" risk-of-bias rating across all included studies, largely attributable to insufficient reporting of allocation concealment; notable inconsistency evidenced by moderate-to-high heterogeneity in the primary PONV and nausea severity outcomes, likely arising from methodological variances in older trials; and imprecision resulting from the relatively small pooled sample size (n=433) and low adverse event rates, which produced wide confidence intervals. A comprehensive summary of findings for all outcomes is detailed in Table 3.

**Table 3:** Summary of Findings and Certainty of Evidence (GRADE) for Primary and Secondary Outcomes. ^a^Downgraded one level due to risk of bias (reporting gaps). ^b^Downgraded one level for inconsistency (conflicting study results or high I^2^). ^c^Downgraded one level for imprecision (wide confidence intervals). ^d^Downgraded two levels for imprecision (single small study or very few events). Malak et al. [9] was not included in this subgroup analysis. GRADE: Grading of Recommendations Assessment, Development and Evaluation; PONV: postoperative nausea and vomiting; RCT: randomised controlled trial; OR: odds ratio; MD: mean difference. Refs. [8,10,11].

Outcome	Participants (Studies)	Relative Effect (95% CI)	Certainty (GRADE)	Comments
Incidence of any PONV (all surgeries)	433 (4 RCTs)	OR 0.74 (0.34 to 1.61)	⨁⨁◯◯ Low^a,b^	Non-significant. Downgraded for bias and heterogeneity (I^2^=68%).
Incidence of PONV (gynaecology subgroup only)	303 (3 RCTs)	OR 0.65 (0.24 to 1.79)	⨁⨁◯◯ Low^a,b^	Sensitivity analysis excluding cholecystectomy. Result remains non-significant with high heterogeneity (I^2^=76%).
Postoperative vomiting	321 (3 RCTs)	OR 1.11 (0.61 to 2.02)	⨁⨁◯◯ Low^a,c^	Non-significant. Downgraded for bias and imprecision.
Rescue antiemetics	321 (3 RCTs)	OR 1.83 (0.95 to 3.52)	⨁⨁◯◯ Low^a,c^	Non-significant. Downgraded for bias and imprecision.
Nausea severity	303 (3 RCTs)	Narrative synthesis	⨁◯◯◯ Very low^a,b^	Conflicting results. One study favoured ondansetron; two found no difference.
Adverse effects (sedation)	247 (2 RCTs)	OR 0.93 (0.43-2.03) and 1.52 (0.25-9.37)	⨁⨁◯◯ Low^a,c^	Non-significant. Downgraded for bias and imprecision (wide CI).
Adverse effects (headache)	130 (1 RCT)	OR 1.00 (0.19 to 5.15)	⨁◯◯◯ Very low^a,d^	Non-significant. Downgraded for bias and very serious imprecision (single study).
Adverse effects (dizziness)	130 (1 RCT)	OR 0.49 (0.09 to 2.76)	⨁◯◯◯ Very low^a,d^	Non-significant. Downgraded for bias and very serious imprecision (single study).
Time to eye-opening	74 (1 RCT)	MD +2.00 min (0.60 to 3.40)	⨁◯◯◯ Very low^a,d^	Significant delay with cyclizine. Downgraded for bias and a single small study.
Time to discharge	74 (1 RCT)	MD -15.00 min (-42.40 to 12.40)	⨁◯◯◯ Very low^a,d^	Non-significant. Downgraded for bias and a single small study.
Pain on injection	112 (1 RCT)	OR 1.81 (0.16 to 20.46)	⨁◯◯◯ Very low^a,d^	Rare events (n=3 total). Downgraded for very serious imprecision.
Patient satisfaction	191 (2 RCTs)	OR 1.19 (0.47-3.02) and 1.29 (0.32-5.23)	⨁⨁◯◯ Low^a,c^	High satisfaction in both groups. Downgraded for bias and imprecision.

Discussion

This systematic review and meta-analysis provides a direct, quantitative comparison of ondansetron and cyclizine, synthesising data from 433 adult surgical patients. Contrary to the historical perception that 5-HT3 antagonists are inherently superior to older antihistamines, our pooled analysis revealed no statistically significant difference between the two agents regarding the incidence of postoperative vomiting, total PONV, or the requirement for rescue antiemetics. While one early study by Watts [10] favoured ondansetron, the aggregate data suggest that both drugs offer comparable prophylactic efficacy in the context of general anaesthesia.

Crucially, our analysis of secondary outcomes challenges the prevailing understanding that cyclizine is unsuitable for ambulatory surgery due to sedation. Although patients receiving cyclizine demonstrated a statistically significant delay in time to eye opening, approximately 2 minutes longer than the ondansetron group, this transient pharmacological effect did not translate into a prolonged hospital stay. There was no significant difference in the total time to discharge, nor were there discrepancies in patient satisfaction scores. Furthermore, the safety profiles were remarkably similar, with no significant increase in adverse events such as headache or dizziness in the cyclizine cohort.

Comparison With Previous Studies

Our findings align with the broader conclusions of recent network meta-analyses, such as Weibel et al. [12], which established that while all major antiemetic classes are superior to placebo, the differences between individual monotherapies are often clinically negligible. However, by focusing exclusively on head-to-head RCTs, our review highlights the heterogeneity that often drives conflicting clinical guidelines. The outlier findings in our analysis stem primarily from Watts [10], who reported a significant benefit for ondansetron. A closer inspection of that trial reveals the use of a distinct "clinical PONV" composite score (nausea score ≥2) rather than pure event rates, which may have amplified the perceived efficacy of ondansetron. In contrast, the more recent trials by Malak et al. [9] and Grimsehl et al. [8] utilised standard event-based reporting and found near-identical outcomes.

Our sensitivity analysis (Figure 4), which excluded laparoscopic cholecystectomy to focus solely on gynaecological procedures, confirmed the robustness of these findings. The lack of statistical difference between the two agents persisted (OR 0.65, 95% CI 0.24-1.79, p=0.41), and heterogeneity remained high (I^2^=76%), further reinforcing that the variance in outcomes is driven by study-specific methodology (e.g., Watts [10]) rather than the surgical population.

Cost-Effectiveness and Clinical Implications

The debate regarding cost-effectiveness is central to this comparison. Both Grimsehl et al. [8] and Watts [10] emphasised the substantial price differential at the time of their publication, with cyclizine representing a "10-fold" cost reduction. While the patent expiry of ondansetron has undoubtedly narrowed this gap in modern practice, the principle of "value-based healthcare" remains relevant. As noted by classic pharmacoeconomic analyses in anaesthesia, when two agents possess equivalent efficacy profiles, the acquisition cost and the burden of side effects become the primary determinants of choice [19]. Our data suggest that the "premium" previously associated with ondansetron does not yield a proportional clinical gain in low-to-moderate risk populations. The results of this study support the continued utility of cyclizine as a viable first-line agent or as a robust component of multimodal prophylaxis. The finding that cyclizine does not delay hospital discharge is particularly relevant for day-case surgery units, where throughput is a key performance indicator. Anaesthetists may be reassured that the anticholinergic sedative effects of cyclizine appear to be short-lived and do not compromise the overall recovery trajectory. However, the trend toward lower rescue antiemetic requirements in the cyclizine group for diagnostic laparoscopy, as observed by Cholwill et al. [11], warrants attention. This may reflect the specific efficacy of antihistamines in managing motion-induced vestibular stimulation, a mechanism often implicated in gynaecological laparoscopic surgery. Consequently, cyclizine may hold a mechanistic advantage in specific surgical subtypes where vestibular stimulation is high, aligning with the consensus that multimodal coverage of different receptors (5-HT3, H1, D2) yields the best protection [1].

Interpretation of the GRADE Assessment

The certainty of evidence was systematically evaluated for all outcomes, resulting in an overall classification of low to very low quality. This grading was primarily influenced by the 'some concerns' risk of bias across included studies, moderate heterogeneity in the primary analysis, and imprecision driven by small sample sizes. Clinically, a low-certainty rating implies that while the current data support the equivalence of cyclizine and ondansetron, these findings represent the best available estimate rather than a definitive conclusion. As such, further research is likely to have an important impact on our confidence in the estimate, and clinicians should remain open to emerging evidence from modern, large-scale pragmatic trials that may provide higher-certainty conclusions.

Limitations

Several limitations must be considered when interpreting these findings. First, the total sample size of 433 participants is relatively small, which limits the power of the analysis to detect rare adverse events. Second, the included studies span a significant timeframe (1996-2017). Anaesthetic practice has evolved considerably during this period, particularly regarding the increased use of propofol TIVA (total intravenous anaesthesia), which possesses intrinsic antiemetic properties [20]. The older studies in our review likely utilised volatile anaesthetics and nitrous oxide, which are highly emetogenic; therefore, the absolute efficacy rates observed in these trials may differ from those seen in modern TIVA-based practice. Finally, the moderate heterogeneity (I^2^=68%) observed in the primary PONV outcome indicates variability in study design and outcome definitions, necessitating a cautious interpretation of the pooled odds ratios. Future investigations should focus on the role of cyclizine within modern multimodal protocols (e.g., combined with dexamethasone) rather than as a monotherapy. Additionally, updated cost-effectiveness analyses are required to reflect current generic pricing structures. Given the potential mechanistic benefit of cyclizine in laparoscopic surgery suggested by our subgroup observations, a large-scale RCT comparing ondansetron versus cyclizine specifically in high-risk laparoscopic populations, controlled for TIVA use, would be valuable to definitively resolve the efficacy debate.

## Conclusions

This systematic review and meta-analysis provides valuable insight into the historical preference for ondansetron over cyclizine in ambulatory surgical procedures. In direct head-to-head comparisons, the 5-HT3 antagonist failed to outperform cyclizine in PONV prophylaxis, preventing postoperative vomiting and reducing the need for rescue antiemetics. This equivalence held true even when the analysis was restricted to high-risk gynaecological laparoscopic procedures. Clinically, these findings challenge the prevailing view that cyclizine is unsuitable for ambulatory settings; while we observed a statistical delay in initial eye-opening, this effect was clinically negligible and did not result in later discharge times or increased adverse events. Economically, given cyclizine's lower acquisition cost, its use as a first-line agent may offer significant value-based savings for healthcare systems without compromising patient care. However, it must be noted that the certainty of this evidence is low, highlighting the need for future pragmatic trials. Considering this limitation and the evolution of anaesthetic practice, future large-scale RCTs should evaluate cyclizine within modern multimodal protocols (e.g., combined with dexamethasone and TIVA) to confirm its efficacy in contemporary settings. Consequently, barring specific contraindications, there is little high-quality evidence to deter clinicians from utilising cyclizine as a primary, cost-efficient option for surgical patients.

## Appendices

**Table 4:** Full search strategies for each database including all keywords, truncation operators, and Boolean logic. Phrases within Quotation marks (“”) allow for exact phrase searching in databases.

MEDLINE	Embase	CENTRAL	CINAHL
(ondansetron[MeSH Terms] OR ondansetron[tiab]) AND (cyclizine[MeSH Terms] OR cyclizine[tiab]) AND (("postoperative nausea and vomiting"[MeSH Terms]) OR "postoperative nausea"[tiab] OR "postoperative vomiting"[tiab] OR PONV[tiab] OR (nausea[tiab] AND postoperative[tiab]) OR (vomiting[tiab] AND postoperative[tiab])) AND (randomized controlled trial[pt] OR controlled clinical trial[pt] OR randomized[tiab] OR placebo[tiab] OR clinical trials as topic[MeSH:noexp] OR randomly[tiab] OR trial[ti])	('ondansetron'/exp OR ondansetron) AND ('cyclizine'/exp OR cyclizine) AND ('postoperative nausea and vomiting'/exp OR 'postoperative nausea and vomiting') AND ('randomized controlled trial'/exp OR 'randomized controlled trial' OR (randomized AND controlled AND ('trial'/exp OR trial)))	(ondansetron) AND (cyclizine) AND (("postoperative nausea and vomiting") OR ("postoperative nausea") OR ("postoperative vomiting") OR PONV OR (postoperative NEAR/3 nausea) OR (postoperative NEAR/3 vomiting))	(ondansetron OR cyclizine) AND (("postoperative nausea and vomiting") OR "postoperative nausea" OR "postoperative vomiting" OR PONV OR (nausea AND postoperative) OR (vomiting AND postoperative)) AND (randomized controlled trial OR controlled clinical trial OR randomized)
